# Targeting the Dynamic Susceptibility Window: Time‐Sensitive Photodynamic Synergy With Antibiotics Against Streptococcus spp

**DOI:** 10.1002/lsm.70116

**Published:** 2026-02-26

**Authors:** Isabella S. Gonçalves, Jennifer M. Soares, Vanderlei S. Bagnato, Kate C. Blanco

**Affiliations:** ^1^ PPG Biotec Federal University of São Carlos São Carlos Brazil; ^2^ São Carlos Institute of Physics University of São Paulo São Carlos Brazil; ^3^ Biomedical Engineering Texas A&M University College Station Texas USA

**Keywords:** antibiotic, antimicrobial resistance, curcumin, photodynamic therapy, *Streptococcus pneumoniae*, *Streptococcus pyogenes*

## Abstract

**Objective:**

Evaluated the antimicrobial efficacy of curcumin‐mediated photodynamic inactivation (PDI) against both sensitive and resistant strains of *Streptococcus pneumoniae* and *Streptococcus pyogenes*, as well as its potential to enhance antibiotic effects (amoxicillin, ceftriaxone, erythromycin).

**Methodology:**

The minimum inhibitory concentrations of antibiotics A, B, and C were evaluated in *S. pneumoniae* and *S. pyogenes* strains using serial microdilution. Additionally, the photodynamic action of curcumin as a photosensitizer was examined in multiwell plates, which were then irradiated with a 450‐nm LED. The effect of the combination over time was determined by colony‐forming units per milliliter, using the determined subinhibitory parameters of antibiotics and PDI.

**Results:**

PDI alone significantly reduced bacterial viability, achieving over 3‐log reductions at optimal conditions (2.5 µM curcumin, 6.4 J/cm^2^). When combined with antibiotics, PDI markedly enhanced bactericidal activity, particularly with β‐lactams, producing up to 2.8 log greater reductions than antibiotics alone. Notably, three consecutive PDI treatments led to substantial MIC reductions, up to 128‐fold for *S. pyogenes*. We identified that the synergistic effect of curcumin‐PDI is highly time‐dependent, revealing a dynamic susceptibility window that can be exploited for enhanced antibiotic action. These results support curcumin‐PDI as both an antimicrobial tool and a sensitizer agent that enhances antibiotic efficacy.

**Conclusion:**

The study highlights that optimal timing of PDI application is key to maximizing its synergistic effects with antibiotics, revealing that PDI is most effective when applied during specific stages of bacterial response to antibiotic stress, potentially redefining adjuvant treatment strategies.

## 1 Introduction

The global rise in antimicrobial resistance (AMR) has increased morbidity, mortality, and healthcare costs, and the World Health Organization has recognized it as one of the top 10 global public health threats [1]. Pathogens such as *Streptococcus pneumoniae* and *Streptococcus pyogenes* have developed resistance mechanisms that undermine the efficacy of many first‐line antibiotics [2, 3]. This resistance arises through mutations in target proteins, the acquisition of resistance genes via horizontal gene transfer, and the overexpression of efflux pumps [4]. Consequently, infections that were once easily treatable now require more toxic or expensive alternatives, and often, therapeutic failure has become increasingly common.

*Streptococcus pneumoniae* is a significant cause of community‐acquired pneumonia, meningitis, and bacteremia, particularly in children, the elderly, and immunocompromised individuals [5]. Resistance in this species has been noted for penicillins, macrolides, and cephalosporins. Similarly, *S. pyogenes*, responsible for pharyngitis, scarlet fever, and necrotizing fasciitis, has shown rising resistance, particularly to macrolides in specific geographic regions [6]. Although *S. pyogenes* remains primarily susceptible to beta‐lactams, treatment failures have been documented, necessitating the exploration of adjunctive therapies [7].

Considering these challenges, PDI has emerged as a promising strategy. PDI relies on the photoactivation of a photosensitizer (PS), which, upon exposure to molecular oxygen and visible light, generates reactive oxygen species (ROS). These ROS, including singlet oxygen and hydroxyl radicals, induce oxidative damage to microbial lipids, proteins, and nucleic acids, leading to irreversible cellular damage and death [8, 9]. Importantly, PDI targets multiple sites within the cell simultaneously, reducing the likelihood of resistance development [10].

Curcumin is obtained by alcohol extraction of Curcuma longa rhizome, yielding a yellow powder composed of three curcuminoids, being 60%–70% curcumin (CUR), 20%–27% demethoxycurcumin (DMC), and 10%–15% bisdemethoxycurcumin (BDMC), which are used as a condiment [11]. Also, the synthetic curcumin exhibits excellent photophysical properties, including a high quantum yield of singlet oxygen, absorption in the visible spectrum (~450 nm), and low dark toxicity [12]. The photophysical characteristics are solvent‐dependent, and in general, their absorption ranges from 408 to 500 nm and their emission from 460 to 560 nm. However, when interacting with hydrophobic surfaces, such as the membrane, the fluorescence shifts toward the blue end of the spectrum [10]. Curcumin degradation photoproducts are described as vanillin, vanillic acid, 4‐vinylguaiacol, ferulic acid, ferulic aldehyde, and 7‐hydroxy‐1‐[(2E)‐3‐ (4‐hydroxy‐3‐methoxyphenyl)prop‐2‐enoyl]−6‐methoxynaphthalene‐2(1H)‐one, which are also photosensitizers, but less photosensitive than curcumin [8]. It preferentially accumulates in Gram‐positive bacteria and accumulates more readily in Gram‐positive organisms due to their peptidoglycan‐rich cell walls [13]. Previous research has shown that curcumin is effective in vitro against various bacterial strains when used in PDI protocols [9, 14, 15, 16, 17, 18, 19, 20, 21]. But comprehensive assessments of its effects on clinically relevant Streptococcus species, particularly in combination with antibiotics, remain scarce [22, 23, 24, 25].

This study aims to fill this gap by evaluating the antibacterial effects of curcumin‐PDI against *S. pneumoniae* and *S. pyogenes*, and by investigating its potential to enhance antibiotic activity and reduce minimum inhibitory concentrations (MICs) following repeated exposure. This combined approach not only addresses bacterial survival but also contributes to controlling persistent streptococcal infections and mitigating resistance mechanisms, potentially reshaping the management of recalcitrant infections. Rather than viewing photodynamic therapy solely as a complementary antimicrobial approach, this study explores the temporal dynamics of the bacterial response to identify optimal windows of vulnerability during antibiotic exposure. The concept of the “window of vulnerability” was explored through temporal analysis of the bacterial response to sequential exposure to antibiotics and PDI, aiming to identify the optimal time to initiate combination therapy. This approach aligns with pharmacodynamic concepts and may contribute to more rational designs of adjuvant therapies. Here, we propose that photodynamic inactivation (PDI) can be optimized not only by dosage, but also by synchronizing its application with critical stages of bacterial stress, thus unveiling a novel pharmacodynamic window of vulnerability.

## 2 Materials and Methods

### 2.1 Bacterial Strains and Culture Conditions

Strains of *S. pneumoniae* (American Type Culture Collection—ATCC 49619 and ATCC 700904) and a clinical isolate of *S. pyogenes* obtained from a patient with pharyngotonsillitis were selected for the study. The clinical isolate of *S. pyogenes* was obtained from a patient diagnosed with pharyngotonsillitis, without additional genotypic identification. The clinical isolate of *S. pyogenes* was obtained from a patient diagnosed with pharyngotonsillitis, without additional genotypic identification. All procedures involving clinical samples were performed in accordance with the ethical guidelines approved by the local Ethics Committee (CAAE 83082018.4.0000.8148; Photodynamic action in the treatment of streptococcal pharyngotonsillitis). All strains were preserved at −80°C in tryptic soy broth (TSB) supplemented with 20% glycerol and 5% defibrinated sheep blood. Before experimental procedures, bacterial cultures were revived in brain‐heart infusion (BHI) broth and incubated at 37°C under 5% CO_2_.

### 2.2 Antimicrobials and MIC Determination

Amoxicillin (AMO), erythromycin (ERY), and ceftriaxone (CEF) were used as representatives of β‐lactam and macrolide antibiotics. MICs were determined by broth microdilution, as outlined in the Clinical and Laboratory Standards Institute (CLSI) guidelines [26]. Serial twofold dilutions of each antibiotic were tested in 96‐well plates in Mueller‐Hinton broth supplemented with 5% sheep blood. Inocula were standardized to 106 CFU/mL. MIC was defined as the lowest concentration showing no visible growth after 24 h at 35°C.

### 2.3 Photodynamic Inactivation

Synthetic curcumin (CUR, EMI Pharma) was prepared as a 5 mM stock solution in ethanol and diluted in phosphate‐buffered saline (PBS) to working concentrations ranging from 0.25 to 5.0 µM. Bacterial suspensions of *S. pneumoniae* and *S. pyogenes* were standardized to 10^8^ colony‐forming units (CFU)/mL in PBS. For each treatment, 250 µL of bacterial suspension was mixed with 250 µL of curcumin solution in 24‐well plates and incubated in the dark for 20 min to allow the photosensitizer to be taken up. Irradiation was performed using a Biotable LED device (450 nm, 55 mW) developed by the Laboratory for Technological Support (LAT) at the São Carlos Institute of Physics, University of São Paulo (IFSC‐USP). The wavelength corresponds to curcumin's absorption peak, and the light dose was set at 6.4 J/cm^2^ delivered over 116 s, aiming for partial bacterial reduction (1–3 log CFU/mL) to assess sublethal effects and potential antibiotic enhancement. Experimental groups included a general control (bacteria in PBS), dark control (5.0 µM CUR without light), light control (irradiation without CUR), and photodynamic treatments at CUR concentrations of 0.25, 0.5, 1.0, 2.5, and 5.0 µM.

After irradiation, samples were serially diluted (1:10) in PBS, plated on blood agar using the drop‐plating method (10 µL/drop), and incubated at 37°C with 5% CO_2_ for 24 h before CFU counting. All assays were performed in biological triplicate. Statistical differences between groups were evaluated by ANOVA followed by Tukey's test (*p* < 0.05). The letters above the graphs indicate significant differences identified by the statistical test, as described in the figure captions.

### 2.4 Time‐Kill Assays for Antibiotics

Time‐kill kinetics were conducted to assess the temporal effects of antibiotics on *S. pneumoniae* (ATCC 49619) and a clinical *S. pyogenes* isolate, following the methods of Keren et al. [27] and Nielsen et al. [28]. Bacterial suspensions were standardized to 10^8^ CFU/mL in BHI broth. Antibiotics were applied at 4× the MIC for AMO and CEF, and at the clinical breakpoint for ERY. For *S. pneumoniae*, all antibiotics were used at 1 µg/mL; for *S. pyogenes*, AMO at 16,384 µg/mL (4× MIC), ERY at 0.25 µg/mL, and CEF at 4 µg/mL. Concentrations were adjusted to achieve measurable antimicrobial activity while accounting for solubility and solvent volume. Samples were incubated at 37°C with 5% CO_2_ and agitation at 400 rpm for 12 h, a duration established through pilot tests that demonstrated near‐total eradication of *S. pneumoniae*. Aliquots were collected at 0, 3, 6, 9, and 12 h, centrifuged, washed in PBS, serially diluted, and plated on blood agar. The plates were incubated at 37°C with 5% CO_2_ for 24 h before enumeration of CFUs.

### 2.5 Combination Therapy

To evaluate the potential interaction between PDI and antibiotics, a time‐kill assay was conducted using *S. pneumoniae* ATCC 49619. Bacterial suspensions were prepared in BHI broth at concentrations of 10^7^ to 10^8^ CFU/mL and incubated with AMO, CEF, or ERY at a concentration of 1 µg/mL. Cultures were incubated at 37°C with 5% CO_2_ and agitation at 400 rpm. At 3‐h intervals (3 and 6 h), aliquots were removed, centrifuged, washed in PBS, and divided into two parts. One part was immediately plated to evaluate the antibiotic‐only effect. The other part was incubated for 20 min in the dark with CUR at 0.5 or 2.5 µM, followed by irradiation using the Biotable LED system (450 nm, 6.4 J/cm^2^), as described previously.

**Figure 1:**
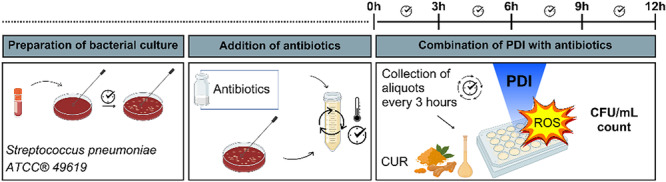
Experimental flow to assess bacterial viability under different treatments. Cultures are treated with antibiotics (AMO, CEF, ERY). Samples at 3 and 6 h are divided: one part to evaluate the antibiotic‐only effect, and another to receive PDI (Photodynamic Inactivation) with curcumin (0.5 or 2.5 µM) and LED irradiation (light dose: 6.4 J/cm^2^). Quantification is by CFU/mL counts.

Samples were then serially diluted, plated on blood agar, and incubated at 37°C with 5% CO_2_ for 24 h. Colony counts (CFU/mL) were used to quantify bacterial viability. The experimental groups included antibiotic‐only treatment (AMO, CEF, ERY), and antibiotic followed by PDI with either 0.5 µM or 2.5 µM curcumin (e.g., AMO + PDI₀.₅, CEF + PDI₂.₅). This study design enables a preliminary assessment of the synergistic or antagonistic effects between the two therapies, which is particularly relevant in the context of rising AMR, where conventional antibiotics alone may be insufficient. Figure 1 illustrates how this experiment was conducted.

### 2.6 Antimicrobial Susceptibility After Repeated PDI Cycles

This study was based on the work of Soares et al. [29], who investigated the effect of five PDI cycles, applied every 6 h, on the MIC of resistant *S. aureus* strains. In the present study, three consecutive PDI cycles were used to investigate the potential of PDI to enhance the susceptibility of *S. pneumoniae* (ATCC 700904) and *S. pyogenes* to antimicrobial agents. This approach aims to explore PDI as an adjuvant strategy to reduce AMR and improve treatment efficacy. The MIC for AMO was previously determined using the standard broth microdilution method, as recommended by the CLSI guidelines (Section 2.2), and served as a reference for this phase of the study.

Bacterial colonies were recovered from frozen stocks and cultivated on blood agar plates for 24 h. Subsequently, colonies were inoculated into PBS, yielding bacterial suspensions at 106 CFU/mL. Each suspension was divided into two groups: one group was incubated with curcumin at 0.5 μM for 20 min, followed by exposure to blue light to perform the first PDI cycle (Cycle 1—C1); the second group was used for baseline MIC determination (MIC₀), without prior PDI treatment. Two different light doses were applied in separate experiments: 6.4 J/cm^2^ and 12.8 J/cm^2^. After C1, aliquots were collected for MIC evaluation (MIC₁), while the remaining suspensions underwent a second (C2) and third (C3) consecutive PDI cycle, each followed by additional MIC determinations (MIC_2_ and MIC_3_, respectively).

After each cycle, suspensions were centrifuged and resuspended in a smaller volume of PBS to concentrate the bacterial load and ensure consistent CFU/mL across all MIC tests. This step was crucial to attribute any observed reduction in MIC values specifically to PDI‐induced changes in bacterial susceptibility, rather than to variations in cell concentration. Following each cycle, suspensions were serially diluted and plated on blood agar to determine CFU/mL, allowing quantitative assessment of bacterial inactivation. All experiments were performed in biological triplicate.

### 2.7 Statistical Analysis

Triplicate experiments (*n* = 9 per group) were analyzed using Shapiro–Wilk normality tests. Parametric data underwent analysis of variance (ANOVA) with Tukey's post hoc tests. Significance was set at *p* < 0.05.

## 3 Results

### 3.1 Antibiotic Susceptibility Profiles

Table 1 shows the MICs of AMO, ERY, and CEF for *S. pneumoniae* and *S. pyogenes*. The MIC values provide an estimate of bacterial susceptibility to antimicrobial agents and serve as a guide for selecting appropriate therapeutic strategies. In this study, monitoring MIC values was also essential for detecting changes in bacterial susceptibility following PDI treatments.

**Table 1:** Minimum inhibitory concentration (MIC) values of *S. pyogenes* and *S. pneumoniae* for the antimicrobials amoxicillin, erythromycin, and ceftriaxone, expressed in μg/mL, and the respective CLSI (Clinical and Laboratory Standards Institute) breakpoints for classifying resistant strains.

Antibiotic	MIC (µg/mL)					
	*S. pneumoniae* (ATCC 49619)		*S. pneumoniae* (ATCC 700904)		*S. pyogenes*	
	Result	Breakpoints	Result	Breakpoints	Result	Breakpoints
Amoxicillin	0.0625	> 1.0	4	> 1.0	8192	> 0.25
Erythromycin	0.0625	> 0.25	16	> 0.25	4096	> 0.25
Ceftriaxone	0.0625	> 2	2	> 2	0.0156	> 0.5

Based on the 2023 clinical breakpoints established by CLSI, *S. pneumoniae* ATCC 49619 was classified as susceptible to all three antibiotics, with MICs of 0.0625 μg/mL for AMO, ERY, and CEF. In contrast, *S. pneumoniae* ATCC 700904 was resistant to all three antibiotics, with MICs of 4 μg/mL (AMO), 16 μg/mL (ERY), and 2 μg/mL (CEF), respectively. The *S. pyogenes* clinical isolate was resistant to AMO (8192 μg/mL) and ERY (4096 μg/mL), but remained susceptible to CEF (0.03125 μg/mL), as defined by the CLSI MIC thresholds.

### 3.2 Photodynamic Inactivation

**Figure 2:**
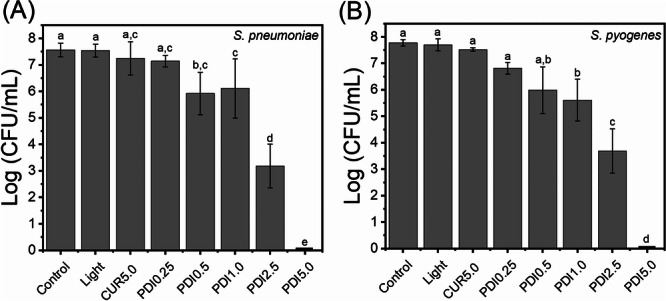
Dose‐dependent bactericidal effect of curcumin‐mediated (PDI) against *S. pneumoniae* (A) and *S. pyogenes* (B). Bacterial viability is expressed as log₁₀ CFU/mL following exposure to increasing concentrations of curcumin (0.25–5.0 µM) under a fixed light dose (6.4 J/cm^2^). Different letters above the bars indicate statistically significant differences among groups; the same letter indicates they are equal (one‐way ANOVA followed by Tukey's test, *p* < 0.05). Bars represent the mean ± standard deviation of three independent experiments.

PDI mediated by CUR, applied without antibiotic association, exhibited a concentration‐dependent bactericidal effect against both *S. pneumoniae* and *S. pyogenes*. As shown in Figure 2, treatments with CUR at 0.25 and 0.5 µg/mL (PDI₀.₂₅ and PDI₀.₅) resulted in little or no reduction in bacterial viability compared to the control. In contrast, PDI₁.₀ and PDI₂.₅ led to moderate (~1.5 log CFU/mL) and substantial (~4.5 log CFU/mL) reductions, respectively. PDI₅.₀ achieved complete eradication of bacterial growth.

These findings confirm a precise dose–response relationship, with higher CUR concentrations enhancing PDI efficacy. The consistency of results across both species suggests that standardized PDI parameters may be applicable within the same bacterial genus. Overall, these outcomes underscore the importance of precise photosensitizer concentration and light dosage for effective photodynamic treatment.

The structural differences between *S. pneumoniae* and *S. pyogenes* may help explain their distinct susceptibility profiles following curcumin‐mediated protein degradation. Unlike *S. pyogenes*, which lacks a prominent capsule, *S. pneumoniae* possesses a thick polysaccharide capsule that serves as a physical barrier, impeding the diffusion of external molecules such as photosensitizers and ROS [30]. This encapsulated architecture not only restricts curcumin penetration but may also hinder the oxidative action of ROS generated during PDI, thereby diminishing its bactericidal efficacy. Maybe this structural constraint was a reason for our observation that *S. pneumoniae* showed lower log reductions in viability at intermediate curcumin concentrations compared to *S. pyogenes*, which responded more robustly even at lower doses. This is a possible explanation reported in the literature, but our study did not experimentally test this mechanism. Thus, the pneumococcal capsule should be considered a limiting factor in PDI effectiveness, requiring optimization of photosensitizer concentration and light dose to overcome this physical and biochemical barrier [30]. Bacterial morphology is essential for their interaction with PS. Depending on the extent of PS absorption by bacteria, it can prevent ROS from reaching their molecular targets. Due to the diffusion of ROS throughout the bacterial cell, the lipophilic characteristic of curcumin facilitates its interaction with the membrane, causing damage by modifying proteins, altering concentration gradients, and disrupting cell wall synthesis, which is sufficient to induce bacterial death. However, these same lipophilic characteristics limit the molecule's ability to cross hydrophilic barriers, such as the bacterial capsule [30, 31, 32, 33, 34, 35].

### 3.3 Time‐Kill Kinetics

The bacterial killing curves for *S. pneumoniae* and *S. pyogenes* were evaluated to assess the time‐dependent reductions induced by AMO, ERY, and CEF. These data served as the basis for investigating the potential effects of combining PDI and antibiotics against the Streptococci. The PDI group was not tested alone because its mechanism of action involves the immediate death of bacterial cells. If complete eradication does not occur, the remaining cells will proliferate, albeit with delays [36, 37].

**Figure 3:**
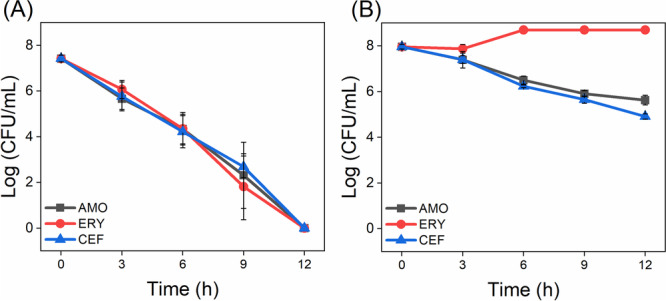
Time‐kill curves of *S. pneumoniae* (A) and *S. pyogenes* (B) following exposure to the antimicrobials: amoxicillin (AMO—0.25 and 16,384 μg/mL), erythromycin (ERY—0.25 μg/mL), and ceftriaxone (CEF—0.25 and 4 μg/mL), with the respective concentrations applied for each bacterial species.

Figure 3 shows the time‐kill curves for *S. pneumoniae* ATCC 49619 (Figure 3A) and a clinical isolate of *S. pyogenes* (Figure 3B) treated with AMO, ERY, and CEF. For *S. pneumoniae*, all antibiotics showed a continuous decrease in viable cells over time, with reductions exceeding 5 log CFU/mL for AMO and CEF, and around 4 log CFU/mL for ERY after 12 h, indicating effective bacterial killing. The bacterial concentration decreased linearly for all antibiotics tested during the assay.

For *S. pyogenes*, ERY treatment maintained a stable bacterial count up to 3 h, followed by a slight increase and stabilization at approximately 105 CFU/mL after 6 h, suggesting possible resistance or persistence. AMO and CEF treatments resulted in a gradual decrease in viable bacteria over time, with reductions of 3–4 log CFU/mL after 12 h. This resistance observed in *S. pyogenes* aligns with the MIC values reported in Table 1. In contrast, AMO and CEF treatments resulted in a gradual decrease in viable bacteria over time, with reductions of 3–4 log CFU/mL after 12 h, indicating continuous but slower efficacy compared to *S. pneumoniae*. These differences highlight the antibiotic‐specific dynamic responses across different bacterial species. In addition, mortality responses are correlated with the mechanisms of action of each antibiotic. Beta‐lactam and cephalosporin antibiotics, such as AMO and CEF, respectively, act by inhibiting bacterial cell wall synthesis during replication, a bactericidal action that leads to cell lysis [13, 38]. Macrolides such as ERY act by inhibiting bacterial protein synthesis by binding to the 50S subunit of the bacterial ribosome, resulting in a bacteriostatic effect [39, 40].

### 3.4 Photodynamic Inactivation Combined With Antibiotics

This study investigated differences in the behavior of *S. pneumoniae* when exposed to antibiotics alone versus after PDI treatment, analyzing the influence of time and photosensitizer concentration on the antimicrobial effectiveness of the combined treatments. Increased effect was defined in this study as a reduction of 2 log₁₀ CFU/mL or more compared to the most effective treatment alone. At the same time, antagonism was characterized by a decrease less than expected from the sum of the individual therapies.

Accordingly, we assessed the most effective combinations of AMO, ERY, and CEF with PDI using two CUR concentrations (0.5 and 2.5 μM) and a fixed light dose of 6.4 J/cm^2^. These parameters were based on prior PDI results (Section 3.2), and the selected CUR concentrations were intended to achieve sublethal doses, enabling partial bacterial inactivation to facilitate behavioral analysis. These findings may serve as a foundation for developing optimized combination‐therapy protocols.

**Figure 4:**
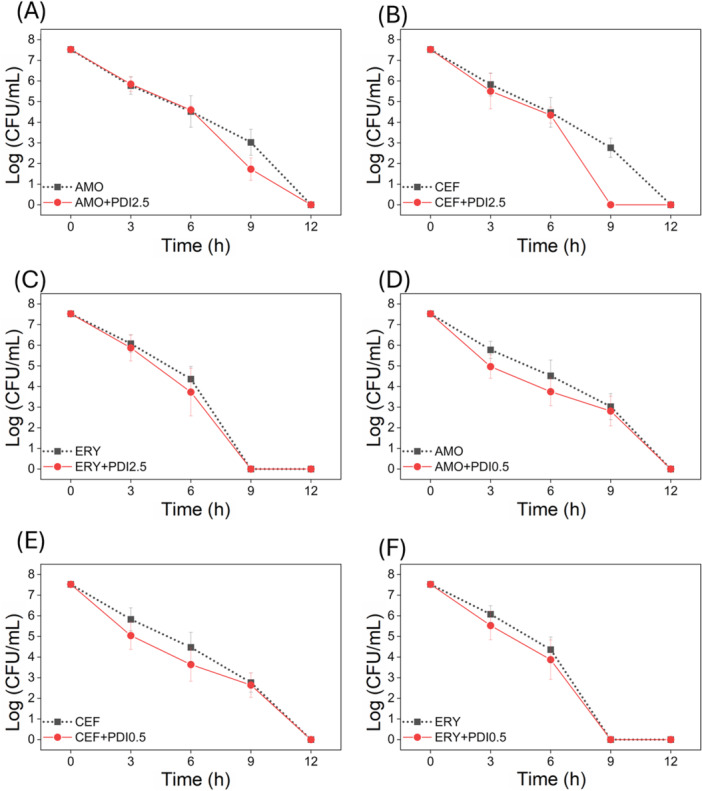
Survival of *S. pneumoniae* (log CFU/mL) over time (h) following treatment. Panels A and D show the effects of amoxicillin (AMO), B and E ceftriaxone (CEF), and C and F erythromycin (ERY). Photodynamic inactivation (PDI) with curcumin (CUR) was applied at 2.5 μM (panels A–C) and 0.5 μM (panels D–F) as a subsequent treatment.

When PDI with 2.5 μM CUR was applied after bacterial incubation with antibiotics, a consistent pattern emerged. At 3 and 6 h of antibiotic exposure, subsequent PDI application did not enhance bacterial reduction. However, at 9 h, the addition of PDI further enhanced the antimicrobial effect compared with antibiotic treatment alone. This pattern was consistent across antibiotics, as shown in Figure 4. An exception was observed with ERY (Figure 4C), which eliminated the bacteria by 9 h, preventing evaluation of the combined effect at that time point.

Figure 4A presents the survival of *S. pneumoniae* over time following incubation with AMO for 12 h and subsequent PDI application (2.5 μM CUR) every 3 h. The concentrations used were 16.384 µg/mL (4× MIC) for AMO, 0.25 µg/mL (4× MIC) for ERY, and 4 µg/mL (4× MIC) for CEF. No significant difference was observed between the combined and monotherapy treatments up to 6 h. However, at 9 h, the PDI application led to a notable 1.3 log CFU/mL reduction compared to AMO alone (AMO + PDI₂.₅). By 12 h, AMO monotherapy was sufficient to eradicate the bacteria. A similar profile was observed for CEF, as shown in Figure 4B. At 3 and 6 h, the combination of CEF with PDI (CEF + PDI₂.₅) showed no significant improvement over CEF alone.

However, after 9 h, this pattern reversed. PDI using 2.5 μM CUR enhanced bacterial reduction when combined with AMO and CEF, by 1.3 and 2.8 log CFU/mL, respectively, while the 0.5 μM CUR condition no longer offered any additional benefit. These findings demonstrate a concentration‐ and time‐dependent behavior of CUR in combination with antibiotics. The most effective result was achieved by combining CEF with 2.5 μM CUR at 9 h, which completely eradicated *S. pneumoniae* 3 h earlier than CEF alone.

The same experiments were repeated using 0.5 μM CUR. Interestingly, the results revealed an inverse pattern compared to the higher CUR concentration. Unlike the 2.5 μM condition, PDI with 0.5 μM CUR enhanced bacterial reduction at earlier time points (3 and 6 h) compared to antibiotics alone. Still, it did not produce significant effects after 9 h, as shown in Figure 4. Figure 4D depicts the bacterial survival (log CFU/mL) over 12 h following AMO exposure and subsequent PDI (0.5 μM CUR) applied every 3 h (AMO + PDI₀.₅). At 3 and 6 h, the combined treatment resulted in approximately 0.8 log reductions in CFU/mL compared with AMO alone. However, at 9 h, no significant difference was observed.

A similar trend was observed for CEF (Figure 4E). The combination CEF + PDI₀.₅ also resulted in a 0.8 log CFU/mL reduction compared to CEF alone at 3 and 6 h, with no significant difference at 9 h. Conversely, for ERY, the combination with PDI₀.₅ showed no significant advantage over ERY monotherapy in the first 6 h (Figure 4F). At 9 h, the bacteria had already been eradicated by ERY alone, rendering further analysis impossible. While reductions in viability were observed, they were limited under the tested subinhibitory conditions. These findings suggest that under these specific parameters, the interaction between PDI and the antibiotic is not strongly synergistic.

It is essential to note that the combination experiments involving antibiotics and PDI were conducted primarily with *S. pneumoniae*. This decision was not due to technical limitations or early eradication in *S. pyogenes*, but rather a deliberate methodological choice based on the response profiles observed in the preliminary assays. *S. pneumoniae* exhibited a more gradual and measurable response to both antibiotic and PDI monotherapies, which enabled the assessment of intermediate bacterial states and the detection of time‐dependent enhanced effects. In contrast, *S. pyogenes* displayed rapid and extensive reductions in viability under the same conditions, making it less suitable for detailed kinetic analysis or for evaluating sequential interventions. Therefore, *S. pneumoniae* was selected as the model organism to explore the temporal dynamics of susceptibility and to define potential pharmacodynamic windows that could guide the development of optimized adjuvant therapies.

### 3.5 Repeated PDI Cycles Reduce MICs

**Figure 5:**
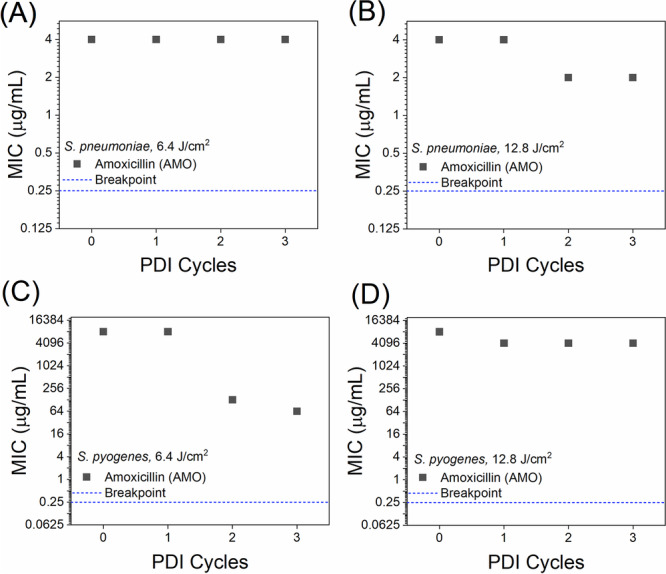
Effects of consecutive photodynamic inactivation (PDI) cycles on the minimum inhibitory concentration (MIC) of amoxicillin (AMO) using two different light doses. Panels show MIC values for *S. pneumoniae* ATCC 700904 after PDI with light doses of 6.4 J/cm^2^ (A) and 12.8 J/cm^2^ (B), and for *S. pyogenes* after PDI with light doses of 6.4 J/cm^2^ (C) and 12.8 J/cm^2^ (D). Breakpoint values established by BRCAST are included to highlight interpretive thresholds for bacterial resistance.

To evaluate the potential of PDI as an adjuvant therapy, we assessed whether repeated exposure to curcumin‐mediated PDI could reduce the MIC of AMO in resistant strains. Figure 5 shows the MIC values of *S. pneumoniae* ATCC 700904 (Figure 5A,B) and *S. pyogenes* (Figure 5C,D) for AMO after each PDI cycle (1, 2, and 3) using 0.5 μM curcumin and two light doses: 6.4 J/cm^2^ (Figure 5A,C) and 12.8 J/cm^2^ (Figure 5B,D). Cycle zero corresponds to the baseline MIC before PDI. For *S. pneumoniae*, at the lower dose (6.4 J/cm^2^), no changes in MIC were observed. In contrast, at the higher dose (12.8 J/cm^2^), the MIC decreased from 4 μg/mL to 2 μg/mL after the second cycle, a twofold decrease.

For *S. pneumoniae*, no change in AMO MIC was observed at the lower light dose (6.4 J/cm^2^). At the higher dose (12.8 J/cm^2^), a twofold reduction in MIC was observed after the second cycle, decreasing from 4 µg/mL to 2 µg/mL. However, no further decrease was observed after the third cycle. Conversely, *S. pyogenes* exhibited a strikingly different behavior. At the lower light dose (6.4 J/cm^2^), AMO MIC decreased from 8192 µg/mL to 64 µg/mL after three PDI cycles, representing a 128‐fold reduction. Surprisingly, the higher light dose (12.8 J/cm^2^) resulted in only a twofold reduction, from 8192 to 4192 µg/mL. These results suggest a species‐specific, dose‐dependent response to PDI, indicating that lower oxidative stress may be more effective at sensitizing *S. pyogenes* to antibiotics. In contrast, higher oxidative levels may activate stress defense mechanisms that attenuate this effect. The data also reinforce the relevance of optimizing PDI parameters for each target organism.

## 4 Discussion

AMR in *S. pneumoniae* and *S. pyogenes* represents a growing clinical concern that underscores the urgent need for alternative or adjunctive strategies. In our study, a clinical isolate of *S. pyogenes* obtained from a patient with pharyngotonsillitis exhibited high‐level resistance to AMO, ERY, and CEF, despite *S. pyogenes* being classically considered susceptible to β‐lactams such as amoxicillin, ampicillin, and oxacillin [41, 42]. Macrolides are commonly prescribed as alternatives for patients with penicillin allergies. However, resistance to this class of antibiotics can emerge rapidly, particularly in regions where macrolides are widely used [42]. Meanwhile, *S. pneumoniae* ATCC 700904 exhibited penicillin resistance, associated with alterations in penicillin‐binding proteins [43], along with emerging macrolide and tetracycline resistance [39]. The World Health Organization currently classifies—pneumoniae as a medium‐priority pathogen for new antimicrobial therapies [43]. Our baseline MIC assessments confirmed that ATCC 700904 is resistant to AMO (MIC = 4 μg/mL), ERY (MIC = 16 μg/mL), and CEF (MIC = 2 μg/mL), while ATCC 49619 remained susceptible (MIC = 0.0625 μg/mL). The *S. pyogenes* isolate exhibited extreme resistance to AMO (MIC = 8192 μg/mL) and ERY (MIC = 4096 μg/mL) yet remained sensitive to CEF (MIC = 0.03125 μg/mL). These resistance phenotypes justified investigating curcumin‐mediated PDI as a potential strategy to restore antibiotic susceptibility or directly inactivate multidrug‐resistant streptococcal strains.

These results align with prior findings, in which CUR concentrations varied from 0.25 to 5.0 µM under a constant light dose of 6.4 J/cm^2^, demonstrating a precise dose–response effect against both *S. pneumoniae* and *S. pyogenes*. At 0.25 µM and 0.5 µM, negligible reductions in viability were observed compared to untreated controls, confirming that CUR's bactericidal activity is strictly photo‐dependent [10, 12]. At 1.0 µM CUR, PDI induced a moderate reduction of approximately 1.5 log₁₀ CFU/mL, whereas 2.5 µM achieved a substantial decrease of ~4.5 log₁₀ CFU/mL. At the highest concentration tested (5.0 µM CUR), complete bacterial eradication was achieved under the applied conditions. These results align with prior findings showing that photoactivated curcumin generates ROS that directly damage bacterial cell walls and DNA [9]. The complete eradication of a multidrug‐resistant *S. pyogenes* isolate further highlights PDI's capacity to overcome antibiotic resistance. High CUR concentrations produce increased ROS flux, which can also alter membrane permeability, potentially facilitating subsequent antibiotic uptake [12, 34, 44].

In this context, we defined an improved effect based on time‐kill assay outcomes. An enhanced effect was considered when the combined treatment of curcumin‐PDI with an antibiotic resulted in a greater reduction in bacterial viability (CFU/mL) than the most effective individual treatment. Specifically, an additional reduction of ≥ 2 log CFU/mL relative to the antibiotic alone at a given time point was used as the threshold for improvement. Guided by these standalone results, we selected 0.5 µM CUR (≈1.5 log₁₀ reduction) and 2.5 µM CUR (≈4.0 log₁₀ reduction) for combination experiments. When PDI was applied after antibiotic exposure, the timing and CUR concentration proved critical. For AMO and CEF, 0.5 µM CUR PDI produced a modest additional 0.8 log₁₀ CFU/mL reduction after 3 h and 6 h compared to antibiotic monotherapy. However, by 9 h, PDI at 0.5 µM showed no further significant effect, likely because the bacterial population was already severely damaged by antibiotics. In contrast, 2.5 µM CUR PDI did not achieved an additional reduction in the first 6 h but produced a pronounced effect at 9 h: AMO + PDI₂.₅ reduced viability by an extra 1.3 log₁₀ CFU/mL, and CEF + PDI₂.₅ by an additional 2.8 log₁₀ CFU/mL, yielding complete eradication of *S. pneumoniae* 3 h earlier than antibiotic alone. The similarity in behavior between AMO and CEF may be attributed to their shared mechanism of action, as both inhibit bacterial cell wall synthesis. In contrast, the combination of ERY and PDI showed no additive effect within the first 6 h, and by 9 h, ERY alone had already eradicated the bacteria, precluding further evaluation. In contrast, erythromycin (ERY), which targets the ribosomal 50S subunit, did not exhibit a better effect with PDI. ERY alone eradicated bacteria by 9 h, eliminating the possibility of further enhancement by PDI. This lack of additive effect may result from its intracellular target and limited ability to disrupt membrane integrity, thereby restricting CUR uptake and ROS accessibility [45, 46].

These results highlight the importance of optimizing both the timing and concentration of the photosensitizer in PDI to maximize its synergistic potential with antibiotics. The observed behaviors suggest that at early stages of antibiotic exposure, lower CUR concentrations may be more effective in enhancing antibacterial activity, possibly due to reduced bacterial resistance mechanisms or compromised cell wall integrity, which allows for better CUR uptake. At later time points, higher concentrations become necessary, perhaps to overcome bacterial adaptation or to produce a sufficient oxidative burst for complete inactivation. Additionally, the variability in outcomes depending on the antibiotic used, particularly the lack of additive effect with ERY, suggests that the mechanism of antibiotic action plays a crucial role in determining its potential impact with PDI. Since ERY targets the bacterial ribosome, rather than the cell wall, it may not facilitate enhanced CUR penetration, thereby limiting the oxidative damage induced by PDI.

We evaluated whether repeated PDI cycles could lower MICs. Three consecutive cycles (at 0.5 µM CUR) were applied without regrowth intervals. For *S. pneumoniae* ATCC 700904, no change in AMO MIC occurred at 6.4 J/cm^2^; however, at 12.8 J/cm^2^, the MIC decreased from 4 µg/mL to 2 µg/mL after the second cycle, indicating a twofold reduction. CEF MICs remained unchanged. Remarkably, *S. pyogenes* exhibited a 128‐fold decrease in AMO MIC (from 8192 to 64 µg/mL) at 6.4 J/cm^2^, whereas at 12.8 J/cm^2^, only a twofold reduction occurred (from 4192 to 2096 µg/mL). This non‐linear dose response likely reflects the balance between optimal ROS generation and activation of bacterial defense mechanisms: lower light doses may produce sufficient ROS to impair envelope integrity and antibiotic uptake without triggering robust protective responses, whereas excessive ROS at higher doses may induce stress responses (efflux pumps, DNA repair) that diminish PDI efficacy [47, 48]. Consecutive low‐dose cycles may maintain bacteria in a vulnerable state, thereby increasing the effectiveness of antibiotics. Population heterogeneity under oxidative stress may also play a role, as subpopulations with distinct susceptibilities emerge [49, 50, 51].

A particularly striking observation in this study was the differential response to repeated PDI cycles between *S. pyogenes* and *S. pneumoniae*. While *S. pneumoniae* exhibited only a modest twofold reduction in the MIC of amoxicillin after three cycles of PDI at the higher light dose (12.8 J/cm^2^), *S. pyogenes* demonstrated a dramatic 128‐fold reduction in MIC using the lower light dose (6.4 J/cm^2^). Interestingly, when the light dose was increased to 12.8 J/cm^2^ for *S. pyogenes*, the MIC reduction was substantially less pronounced (only twofold). This inverse dose‐dependent behavior suggests that excessive oxidative stress may activate bacterial defense mechanisms, such as DNA repair systems, efflux pumps, or oxidative stress regulators, ultimately counteracting the sensitizing effects of PDI. Thus, mild oxidative perturbation may be more effective in shifting the physiological state of the bacterium toward increased susceptibility, while higher oxidative insults may instead trigger protective responses.

The marked response of *S. pyogenes* to low‐dose PDI also reflects its intrinsic susceptibility to ROS and membrane permeabilization. Lacking a thick polysaccharide capsule, *S. pyogenes* may allow easier penetration of curcumin and diffusion of ROS into the cytoplasm, enabling more effective disruption of membrane integrity and intracellular targets even at low light doses. Moreover, the absence of capsule‐associated protection means that PDI‐induced damage accumulates rapidly, possibly enhancing antibiotic uptake and amplifying the overall antimicrobial effect. In contrast, *S. pneumoniae*'s encapsulated architecture likely restricts early curcumin access and ROS diffusion, requiring higher light doses to achieve comparable effects. Even at those doses, the induction of resistance mechanisms may limit the overall MIC reduction.

Literature studies indicate that curcumin can generate ROS upon activation in the blue region of the electromagnetic spectrum, thereby effectively inhibiting the growth of *Streptococcus mutans* in both planktonic cultures and biofilms [52, 53]. Although further investigation is needed, existing research indicates that, due to its fat‐soluble properties, curcumin interacts with cell membranes, altering local fluidity [32, 34, 54]. This change affects both intra‐ and extracellular communication by disrupting and altering gramicidin A channels [54, 55].

Additionally, a key physiological distinction lies in *S. pneumoniae*'s endogenous production of hydrogen peroxide (H_2_O_2_), a byproduct of its aerobic metabolism. This constitutive oxidative environment may precondition *S. pneumoniae* to moderate ROS levels, activating antioxidant defenses such as thiol peroxidases, superoxide dismutases, and DNA repair enzymes [56]. These adaptations likely confer resilience to PDI‐induced oxidative stress, requiring more intense treatment conditions to overcome bacterial defenses. In contrast, *S. pyogenes* does not naturally produce H_2_O_2_ and may lack similarly robust detoxification systems, making it more vulnerable to exogenous ROS generated by photodynamic action. This contrast in redox physiology may explain the more substantial sensitization observed in *S. pyogenes* after repeated low‐dose PDI, further reinforcing the need to tailor PDI protocols according to species‐specific stress responses.

Although the reduction in AMO MIC after repeated cycles of PDI did not reach the clinical breakpoint for susceptibility, the observed decrease demonstrates PDI's potential to modulate bacterial susceptibility. This effect may be associated with increased membrane permeability induced by ROS, enhancing antibiotic uptake. Additionally, PDI may impact gene expression related to virulence and resistance, influencing the overall response of the bacterial population to antimicrobial treatment [50, 51, 56, 57, 58]. Recent findings from a nationwide surveillance study in Japan indicate that strains of *S. pneumoniae* with low MICs suggest that the serum and lung levels that can be achieved are adequate for effective treatment [59]. These findings highlight a dual mechanism by which PDI not only inactivates bacteria through ROS‐mediated damage but also sensitizes them to antibiotics, suggesting that PDI may serve as a promising adjuvant strategy for managing AMR.

Intrinsic species differences further modulated outcomes: *S. pneumoniae*'s thick polysaccharide capsule can impede CUR penetration and ROS diffusion at low doses [30], and its robust oxidative stress defenses, including endogenous hydrogen peroxide production, may neutralize moderate ROS levels, requiring higher light energy to overcome antioxidant defenses. In contrast, *S. pyogenes*, lacking such a capsule, proved highly susceptible to low‐dose PDI. Although AMO MICs did not fall below clinical breakpoints, the observed two‐ and 128‐fold reductions demonstrate PDI's potential to sensitize highly resistant strains, either by increasing membrane permeability or by modulating expression of resistance‐ and virulence‐associated genes [17, 57, 58, 60]. Consequently, PDI may reduce the antibiotic concentrations required for effective treatment.

Taken together, these findings demonstrate that CUR‐mediated PDI can directly inactivate multidrug‐resistant *S. pneumoniae* and *S. pyogenes*, synergize with β‐lactam antibiotics in a time‐ and concentration‐dependent manner, and lower AMO MICs through consecutive treatment cycles. Achieving this requires careful optimization of CUR concentration, light dose, and timing relative to antibiotic exposure. Specifically, 0.5 µM CUR PDI enhances antibiotic activity at early time points (3–6 h), while 2.5 µM CUR PDI is most effective at later time points (9 h). Species‐specific traits—such as capsule thickness, oxidative stress defenses, and innate resistance mechanisms—have a profound influence on PDI outcomes. For clinical translation, further steps include characterizing CUR pharmacokinetics in vivo, optimizing light delivery (wavelength, irradiance, tissue penetration), and identifying potential antagonistic interactions (e.g., ROS‐mediated antibiotic degradation). In vivo studies must confirm that combined PDI and antibiotic therapy reduces the infection burden, limits the emergence of resistance, and preserves host tissue integrity.

Future research should explore additional antibiotic classes, diverse bacterial species (including Gram‐negative pathogens), and improved PS formulations (e.g., nanoparticle‐encapsulated CUR) to enhance solubility and target delivery. Elucidating PDI‐induced transcriptomic and proteomic changes will clarify mechanisms underlying sensitization and resistance modulation. These efforts will inform standardized PDI protocols tailored to specific antibiotic regimens, ultimately broadening the antimicrobial toolkit against resistant pathogens. In conclusion, curcumin‐mediated PDI represents a promising adjuvant to conventional antimicrobials, capable of directly reducing resistant bacterial populations, enhancing antibiotic efficacy, and lowering antibiotic MICs. Integrating PDI into existing treatment paradigms may extend the clinical lifespan of current antibiotics and help combat the escalating threat of AMR.

Our results indicate that the curcumin‐PDI effect is strongly dependent on the bacterial physiological state, which evolves during antibiotic exposure. This supports the hypothesis that there is a temporal window during which bacteria, although damaged but not yet eradicated, are more susceptible to oxidative stress. Such insights underscore the importance of integrating microbiological dynamics into the pharmacological planning of combination therapies. These results suggest that the timing of PDI‐antibiotic treatment could be adjusted based on infection kinetics, opening up new possibilities for dynamic treatment protocols in resistant infections.

## 5 Conclusions

This study demonstrates that curcumin‐mediated PDI effectively reduces bacterial viability and enhances the activity of conventional antibiotics against *S. pneumoniae* and *S. pyogenes*. PDI does not act uniformly and is partially restored in resistant strains, highlighting its potential as an adjuvant strategy in antimicrobial therapy.

The outcomes varied depending on the antibiotic used and the experimental conditions, such as curcumin concentration, light dose, and timing of application. Combinations with β‐lactam antibiotics showed greater effect, likely due to increased membrane permeability induced by ROS. In contrast, no additional effect was observed with erythromycin, suggesting that the mechanism of antibiotic action plays a critical role in PDI interactions.

These results highlight the importance of optimizing key parameters to achieve consistent synergistic effects. While PDI does not act uniformly across all antibiotics, it represents a promising complementary approach to enhance current treatments and address the growing problem of AMR. Future studies should focus on mechanistic analyses and in vivo validation to support the clinical translation of PDI‐antibiotic combinations.

## Author Contributions

Conceptualization: Jennifer M. Soares, Kate C. Blanco, and Vanderlei S. Bagnato. Methodology: Isabella S. Gonçalves. Formal analysis: Isabella S. Gonçalves and Jennifer M. Soares. Writing – original draft preparation: Isabella S. Gonçalves, Jennifer M. Soares, Kate C. Blanco, and Vanderlei S. Bagnato. Writing – review and editing: Isabella S. Gonçalves, Jennifer M. Soares, Kate C. Blanco, and Vanderlei S. Bagnato. Supervision: Kate C. Blanco and Vanderlei S. Bagnato. Project administration: Vanderlei S. Bagnato. Funding acquisition: Vanderlei S. Bagnato. All authors have read and agreed to the published version of the manuscript.

## Conflicts of Interest

The authors declare no conflicts of interest.

## Data Availability

The data presented in this study are available on request from the corresponding authors.
